# Study protocol for a multicenter prospective randomized-controlled trial to test a systematic remote and app-based prehabilitation program in comparison to education-only before major surgery: the LUMOS Study

**DOI:** 10.1186/s13063-026-09977-w

**Published:** 2026-09-10

**Authors:** Svenja Sliwinski, Franziska J. Stickl, S. Fatima Faqar-Uz-Zaman, Charlotte Detemble, Colin Maurer, Ramona Moldovan Diaremes, Alexander Buia, Marco Niedergethmann, Christoph Reissfelder, Gregor A. Stavrou, Rizky Widyaningsih, Dora Zmuc, Armin Wiegering, Johannes Fleckenstein, Andreas A. Schnitzbauer

**Affiliations:** 1 Department for General, Visceral, Transplant and Thoracic Surgery, Goethe University Frankfurt/Main, University Hospital Frankfurt, Frankfurt/Main, Germany; 2 Ruhr Universität Bochum, Klinik für Chirurgie, Knappschaft Kliniken Universitätsklinikum Bochum, Bochum, Germany; 3 Department of General, Visceral and Thoracic Surgery, Asklepios Clinic Langen, Langen, Germany; 4 Alfried Krupp Krankenhaus Essen, Klinik für Allgemein- und Viszeralchirurgie, Essen, Germany; 5 Department of Surgery, Medical Faculty Mannheim, University Hospital Mannheim, Heidelberg University, Mannheim, Germany; 6 Department of General, Visceral, and Oncological Surgery, Klinikum Saarbrücken, Saarbrücken, Germany; 7 MCL Medical Center Ljubljana, Ljubljana, Slovenia; 8 Department of Sports Medicine Exercise Physiology, Institute of Sports Sciences, Goethe University Frankfurt, Frankfurt am Main, Germany; 9 Department of Pain Medicine, Klinikum Landsberg am Lech, Landsberg am Lech, Germany

**Keywords:** Prehabilitation, Major surgery, Perioperative management, Digitalization, Neoadjuvant therapy

## Abstract

**Background:**

Recent studies have demonstrated significant benefits associated with prehabilitation prior to major surgery. However, digital solutions in this domain are currently lacking. Our previous study, the Protego Maxima trial, reported positively assessed usability, validity, and safety of a prehabilitation application. In this randomized-controlled multicenter study, we aim to ascertain whether a digital prehabilitation program can reduce perioperative morbidity and costs in patients undergoing major surgery.

**Methods:**

The LUMOS trial is a multicenter prospective randomized-controlled trial comparing a 3-to-6-week interventional digital prehabilitation program with a digitized informational app, against a control group receiving only the informational application. The trial seeks to enroll 382 participants who will be block-randomized based on diagnosis into either the intervention or control group. The primary endpoint is the reduction of 90-day morbidity from 60 to 45% (Dindo–Clavien I–V). Secondary objectives include the 1-year overall survival, quality of life, length of hospital stay (including ICU, IMC, and general ward), readmission rates, compliance, and program adherence, product malfunction, vigilance reporting, improvement of the 6-min walking test by at least 20%, and hospital cost assessment.

**Discussion:**

The LUMOS trial aims to evaluate the effectiveness of a digital prehabilitation program compared to an informational app only, prior to major surgery. The objective is to demonstrate the significant value of a digitized remote prehabilitation program in reducing perioperative morbidity, as well as addressing cost considerations.

**Trial registration:**

Trial registered in the European Database on Medical Devices (EUDAMED) (CIV-24-02-046162) and German Clinical Trial Registry (DRKS00033612, 13.05.2024), DMIDS-Number: 00015083, https://drks.de/search/en/trial/DRKS00033612. Goethe University Frankfurt: 024-1681-MDR/MPDG-zuständig multizentrisch, 06.05.2024.

**Supplementary Information:**

The online version contains supplementary material available at https://doi.org/10.1186/s13063-026-09977-w.

## Administrative information

**Table:** 

Title {1}	Study protocol for a multicenter prospective randomized-controlled trial to test a systematic remote and app-based prehabilitation program in comparison to education-only before major surgery: the LUMOS Study
Trial registration {2a and 2b}	European Database on Medical Devices (EUDAMED: CIV-24-02−046162) and German Clinical Trial Registry (DRKS00033612, 13.05.2024), DMIDS-Number: 00015083. Goethe University Frankfurt: 024–1681-MDR/MPDG-zuständig multizentrisch, 06.05.2024
Protocol version {3}	Lumos Clinical Trial Protocol V1.3; June 7, 2024
Funding {4}	The trial is funded by the Distr@l program of the Hessen Ministry for Digital Strategy & Development with a total sum of 894,000€ over 2 years of which 65% are covered by the fund and the gap by the Capreolos GmbH. More information is available at Entwicklung und Validierung der Prehab-App (lidia-hessen.de) (www.lidia-hessen.de/projekte-entdecken/entwicklung-und-validierung-der-prehab-app/)
Author details {5a}	Svenja Sliwinski^1^, Franziska J. Stickl^1^, S. Fatima Faqar-Uz-Zaman^1^, Charlotte Detemble^2^, Colin Maurer^1^, Ramona Moldovan Diaremes^1^, Alexander Buia^3^, Marco Niedergethmann^4^, Christoph Reissfelder^5^, Gregor A. Stavrou^6^, Rizky Widyaningsih^6^, Dora Zmuc^7^, Armin Wiegering^1^, Johannes Fleckenstein^8,9^ and Andreas A. Schnitzbauer^1,2^ ^1^University Hospital Frankfurt, Goethe University Frankfurt/Main, Department for General, Visceral, Transplant and Thoracic Surgery, Frankfurt/Main, Germany ^2^Ruhr Universität Bochum, Klinik für Chirurgie, Knappschaft Kliniken Universitätsklinikum Bochum, Germany ^3^Asklepios Clinic Langen, Department of General, Visceral and Thoracic Surgery, Langen, Germany ^4^Alfried Krupp Krankenhaus Essen, Klinik für Allgemein- und Viszeralchirurgie ^5^University Hospital Mannheim, Department of Surgery, Medical Faculty Mannheim, Heidelberg University, Mannheim, Germany ^6^Klinikum Saarbrücken, Department of General, Visceral, and Oncological Surgery, Saarbrücken, Germany ^7^MCL Medical Center Ljubljana, Ljubljana, Slovenia ^8^Goethe University Frankfurt, Institute of Sports Sciences, Department of Sports Medicine Exercise Physiology, Frankfurt am Main ^9^Klinikum Landsberg am Lech, Department of Pain Medicine, Landsberg am Lech, Germany
Name and contact information for the trial sponsor {5b}	Svenja Sliwinski, MD University Hospital Frankfurt Goethe University Frankfurt/Main Department for General, Visceral, Transplant and Thoracic Surgery Frankfurt/Main, Germany svenja.sliwinski@ukffm.de
Role of sponsor {5c}	The sponsor manages the study and the writing of the paper for publication

## Introduction

### Background and rationale {6a}

Surgery ranks among the top three causes of mortality in hospital settings [1]. Complications following surgical procedures impact up to 60% of patients, exerting both short- and long-term repercussions, including heightened morbidity, mortality, and prolonged hospitalization [2, 3]. These adverse events significantly influence patient satisfaction and overall experience. Notably, the risk of complications is influenced by various factors, including cancer diagnosis, age, frailty, and comorbidities that may be modifiable and optimized before surgery [4–7]. With nearly 40% of individuals in high-income countries aged over 65 years, and given that the average age of cancer and other condition diagnoses align, a considerable portion of patients with these conditions will necessitate surgical interventions in the coming years [8]. Evidence indicates that appropriate exercise interventions can notably enhance a patient’s physical fitness before surgery without exacerbating the risk of mortality from the underlying disease (3–6 weeks) [9–12]. Prehabilitation, a burgeoning field in medicine, focuses on improving individual patients’ physical fitness, therefore enabling them to adequately prepare for surgery. The preoperative period offers a critical window of opportunity wherein patients can enhance their overall physical condition, thus significantly impacting their outcomes. Person-centered prehabilitation of modifiable risk factors places the patient at the forefront of their perioperative journey. Recent randomized-controlled trials from Spain and the Netherlands have demonstrated the benefits of structured preoperative exercise programs, including reduced complications and accelerated recovery [13, 14]. For instance, a 4-week multimodal prehabilitation program significantly reduced severe complications in colorectal cancer patients compared to standard care. Frail patients undergoing elective major abdominal surgery particularly benefited from structured prehabilitation, experiencing improved aerobic capacity and reduced postoperative complications.

Our research group, in collaboration with the German Association for General and Visceral Surgery, conducted a scoping review to evaluate the potential and structured approach to prehabilitation [7]. We concluded that structured data assessment is essential for establishing comparability and enabling global implementation. Considering ongoing healthcare workforce shortages, lack of prehabilitation reimbursement, and the increasing availability of digital therapies, there is a compelling rationale for implementing remote, home-based, yet supervised and connected prehabilitation programs before major surgeries [15].

Our hypothesis is that patients undergoing structured prehabilitation with The Prehab App as a digitized individualized prehabilitation tool before major surgery will experience a reduction in the overall complication rate from 60 to 45%.

### Objectives {7}

#### Primary endpoints

The primary objective of this study is to analyze the safety and efficacy of The Prehab App before major surgical procedures. We hypothesize that the rate of postoperative morbidity can be reduced from 60 to 45%. The primary endpoint is the occurrence of any postoperative complication within 90 days after surgery according to the Clavien–Dindo classification (grades I–V).

#### Secondary endpoints

Secondary endpoints of this study are the 1-year overall survival, the compliance and adherence to The Prehab App, the hospital cost effects of The Prehab App (readmission, length of hospital stay, reduction of cost), the quality of life, improvement of the 6-min walking test, the number of device failures, the vigilance of the device before surgery, and to identify patterns of repair, vascular remodeling, tumor microenvironment, and mitochondrial health in a future step.

### Trial design {8}

The LUMOS trial is a prospective and randomized-controlled trial. Here, a 3-to-6-week interventional digital prehabilitation program (The Prehab App and medical guidance) with a digitized informational app (Ready4Op) will be compared against a control group receiving only the informational application as a parallel group. The patient allocation ratio is 1:1. The framework of the trial is superiority.

#### Study population

**Fig. 1:**
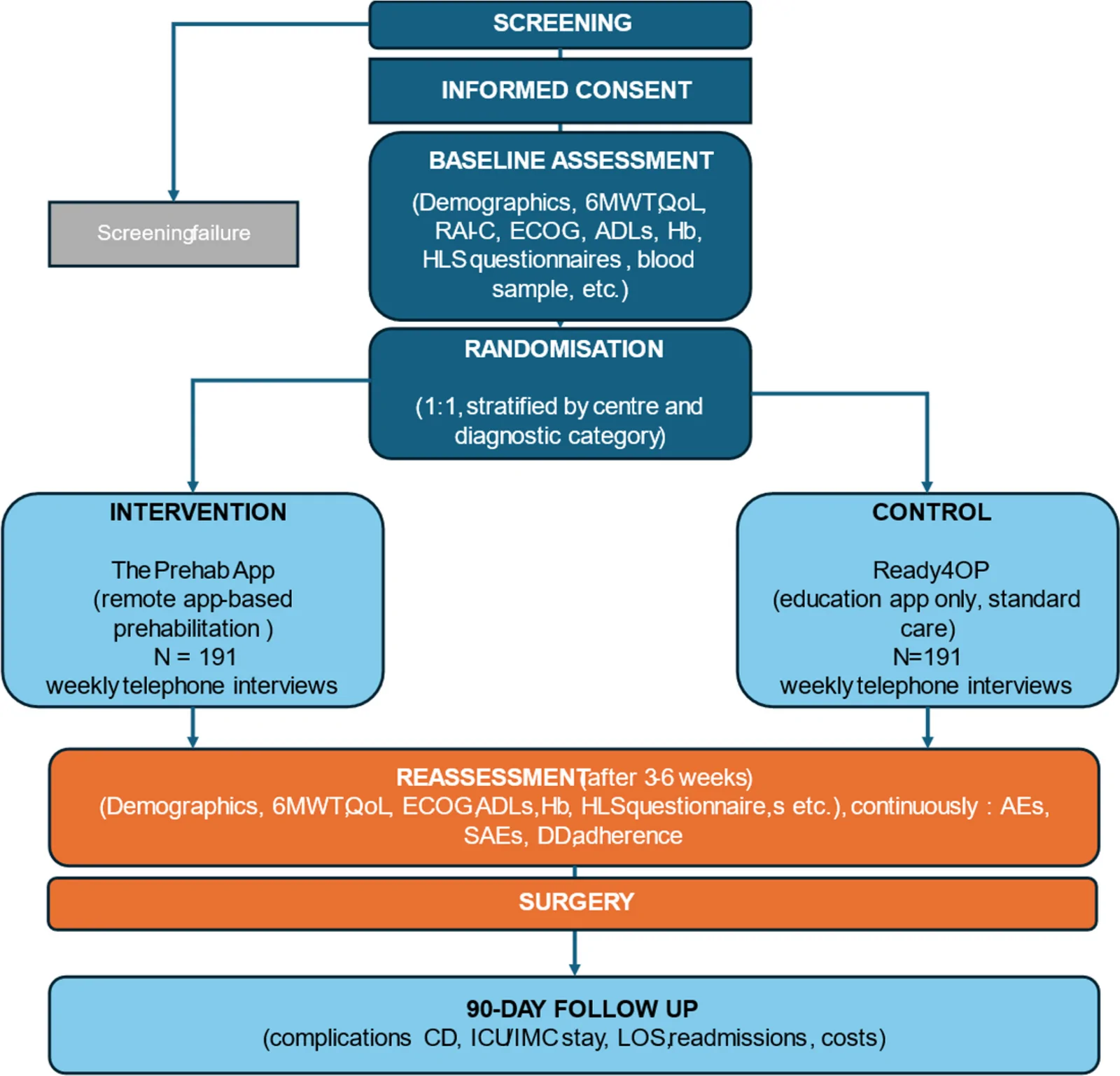
Study flow diagram of the LUMOS trial

The study population will comprise 382 patients eligible for a major surgical procedure, possessing the mental, physical, and medical capacity to engage in a remote prehabilitation program. The program will last 3 weeks in cases where a cancer diagnosis does not necessitate neoadjuvant treatment or any other justified waiting period. For patients with a non-malignant diagnosis or those undergoing neoadjuvant therapy, the duration of the prehabilitation program will extend to 6 weeks. One hundred ninety-one patients will be randomized into the study arm using The Prehab App and 191 patients will be the controls using the Ready4Op App (Fig. 1).

## Methods: participants, interventions, and outcomes

### Study setting {9}

Patient recruitment is taking place at 6 trial centers in Germany; the hospitals vary between university hospitals and maximum care hospitals. All participating centers are highly experienced in major surgeries. The list of study sites can be obtained upon request from the corresponding author or under https://drks.de/search/de/trial/DRKS00033612. Reporting was done according to the SPIRIT guidelines (supplements 2) [16].

### Eligibility criteria {10}

The eligibility criteria for study centers are German hospitals that are experienced in major surgeries. The principal investigators and other investigators need to be doctors in surgery at the corresponding hospital.

#### Inclusion criteria

Patients must meet the following criteria to be eligible for the study:

The patient provides written informed consent, is able to understand the content of the study, understands the requirements for follow-up visits, and is willing to complete the questionnaires and provide the required information at follow-up visits.

The eligibility criteria encompass the following: (1) individuals aged ≥18 years, (2) participants possessing the cognitive capacity to comprehend the assigned task and provide written informed consent, (3) patients undergoing elective major surgeries including gastrointestinal resection, resection of the hepatobiliary and pancreatic system, endocrine glands, lung or bronchus, splenectomy, abdominal wall hernia repair, urological or gynecological resections, or vascular surgery devoid of cardiovascular procedures. The surgery can hereby be performed open, laparoscopic, or robotic assisted. Corresponding Operation and Procedure Classification System (OPS) codes are delineated in the supplemental material (Table S1).

Patients are only allowed to enter the trial if they provide their written informed consent and if the responsible investigator has verified that all selection criteria are met.

#### Exclusion criteria

If the patients meet any of the following criteria at the screening visit, they will not be eligible for the study:

Primary exclusion criteria involve (1) pregnancy or lactation, (2) inability to comprehend or engage in the task, (3) acute cardiovascular or pulmonary ailments, or (4) acute non-cardiopulmonary disorders susceptible to or exacerbated by exercise performance (e.g., infection, renal failure).

### Who will take informed consent? {26a}

Patients slated for elective major surgery will undergo eligibility screening conducted by the study team within outpatient departments. Furthermore, all physicians will receive training and orientation regarding the recruitment process to enhance recruitment efficacy. Eligible patients will be informed about the study by trained study physicians or principal investigators delegated by the participating center. Written informed consent will be obtained by a trained investigator prior to any study-specific procedures.

### Additional consent provisions for collection and use of participant data and biological specimens {26b}

Furthermore, given the incomplete understanding of the molecular mechanisms and effects of prehabilitation, patients will be asked to consent to tissue research (using resection specimens). This research aims to elucidate mechanisms of vascular repair, microenvironmental changes, and mitochondrial health. These endeavors are considered ancillary projects necessitating supplementary funding. At present, consent will solely be sought for participation in these ancillary projects, in addition to the regular informed consent for the clinical trial.

## Interventions

### Explanation for the choice of comparators {6b}

The comparator used is the digitized informational app (Ready4Op). Ready4Op version 1 is a classic digital textbook without any interventional approaches to the patient. There is a basic demographic survey of height, weight, BMI, diet preferences, and fitness level of each patient before prompted to the content area which triggers individualization of the content. The content consists of 9 categories of which the 6 freely available consist of the prehab-specific content. In more than 50 subchapters, the patients receive valuable evidence-based information about the perioperative phase and can use the digital textbook as a knowledge base. There is no exchange or storage of data on a server, the demographics remain on the individual smartphone. There will be no direct readout. To better understand what the patients gained from the application, a set of standard PROMs will be documented to compare the results to the interventional group described above. The choice of comparator is the minimum recommended information patients should receive before major surgery. Experience and data show that this information often comes much too short and therefore is more than standard in an average preoperative preparation. The resource-sparing information on perioperative options to improve the individual situation before major surgeries is standard of care and does not deviate from common practice.

### Intervention description {11a}

#### Prehab App

**Fig. 2:**
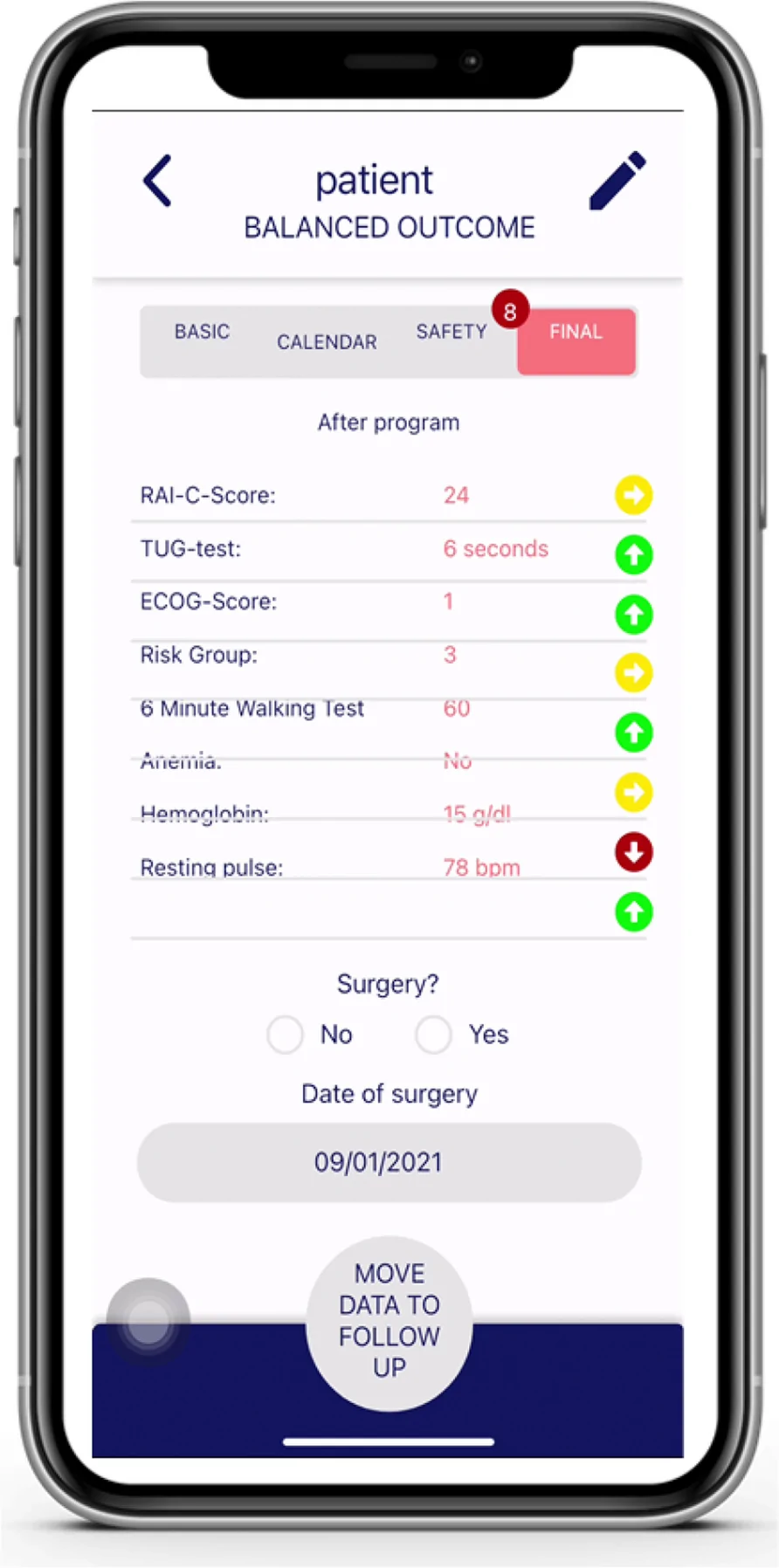
Schematic layout of the app surface with exemplary risk scoring

The Prehab App is a medical software designed to create individually tailored aerobic interval exercise training programs for patients aged 18 years and older requiring major elective surgery. It will be a medical device class IIa and is currently developed by Capreolos GmbH, a spin-off company of the Goethe University Frankfurt/Main. The app encompasses a structured risk assessment (Fig. 2), based on demographic data and individual risk factors. The patient will then be stratified into a specific risk group utilizing the Karvonen method [17]. A blood sampling takes place.

The physician assesses the patient’s risk group using a desktop computer or the app. Then, the standalone software generates a personalized risk-based endurance exercise program and calculates the target heart rate (HR), incorporating maximum heart rate, resting pulse, and individual risk factors. Afterwards, the personalized training program is installed on the patients’ smartphones, where they will start with a 3–6-week aerobic interval training regimen (Fig. 1).

Prior to exercising, patients receive structured guidance as per the instruction manual and are screened for contraindications such as acute respiratory infections.

Before each session, patients complete a baseline QLQC-30 survey and a 6-min walking test. Each interval session lasts 47 min, including 5 min of warm-up and 5 min of cool-down, and will be performed 3 to 4 times. The training consists of alternating 2-min intense and 3-min less intense intervals. Heart rate and distance will be monitored during both the initial and final 6-min walking tests, as well as during individual training sessions. This monitoring will be facilitated via an Application Programming Interface (API) that interfaces with a smartwatch worn on either the right or left wrist tracking heart rate and distance.

Patients follow instructions on their wearable to stay within their threshold levels displayed on the screen, indicated by green (within) or red (outside) coloration. Vibration alerts signal training outside defined thresholds. Following the 6-MWT or exercise, patients are directed to a summary page to review data, and patient-reported outcome measures (PROMs) regarding symptoms experienced during exercise are collected. The Prehab program ends with a final 6-MWT and QLQC-30 survey. Then a second assessment calculating the risk scoring again by a physician follows directly before surgery. A second blood sampling takes place. The intervention results are then correlated with the 90-day follow-up data. The 90-day follow-up is undertaken as a telephone interview.

For the patient, no other equipment than a smartphone having The Prehab App installed and a smartwatch is needed. The app works with all common smartwatch and smartphone software on the market (e.g., iOS, Android).

The software facilitates home-based, remote exercise sessions, which can occur outdoors in nature or indoors on a treadmill, indoor cycle, or indoor track, at any time of day. Patients have autonomy in selecting their exercise environment, including factors such as lighting, noise level, terrain, location, and timing, to suit their schedule and preferences.

The comparator group is using the Ready4Op App which gives information generally provided preoperatively.

After completing the Prehab program via the app as described above, upon return to the hospital and prior to surgery, a second risk assessment is conducted. Here, delta values are calculated, indicating improvements, a stable condition, or a decline in assessed points, serving as decision support for continuing with the operation or assigning alternative therapies.

Throughout the program, a safety check is initiated on both the physician and patient displays according to an algorithm derived from the software’s risk management. To track app or device failure, safety, and misuse scenarios, testing the app’s alarm settings will be conducted at the same time. A 24/7 emergency hotline connects patients to the treating hospital. Physicians can monitor the exercise data of each patient in their department to track progress, while individual patients can review their progress within the app.

#### Assessments during the treatment phase

Intervention visits are performed weekly during the prehabilitation period and contain the evaluation of PROMs, compliance, safety audit, and retrieval of digitized data (V2–V7) (Table 1). Blood samples will be collected before the first and after the last interval training to further evaluate the safety of app-based exercise training. Additionally, the validity of the Karvonen method, including risk adjustments based on defined risk factors, will be assessed and correlated with the occurrence of clinical symptoms. To do so, 1–3 days before surgery, reassessment is conducted by performing a 6-min walking test and by evaluating patient history, risk factors, anemia, and heart rate (V5/V8).

**Table 1:**
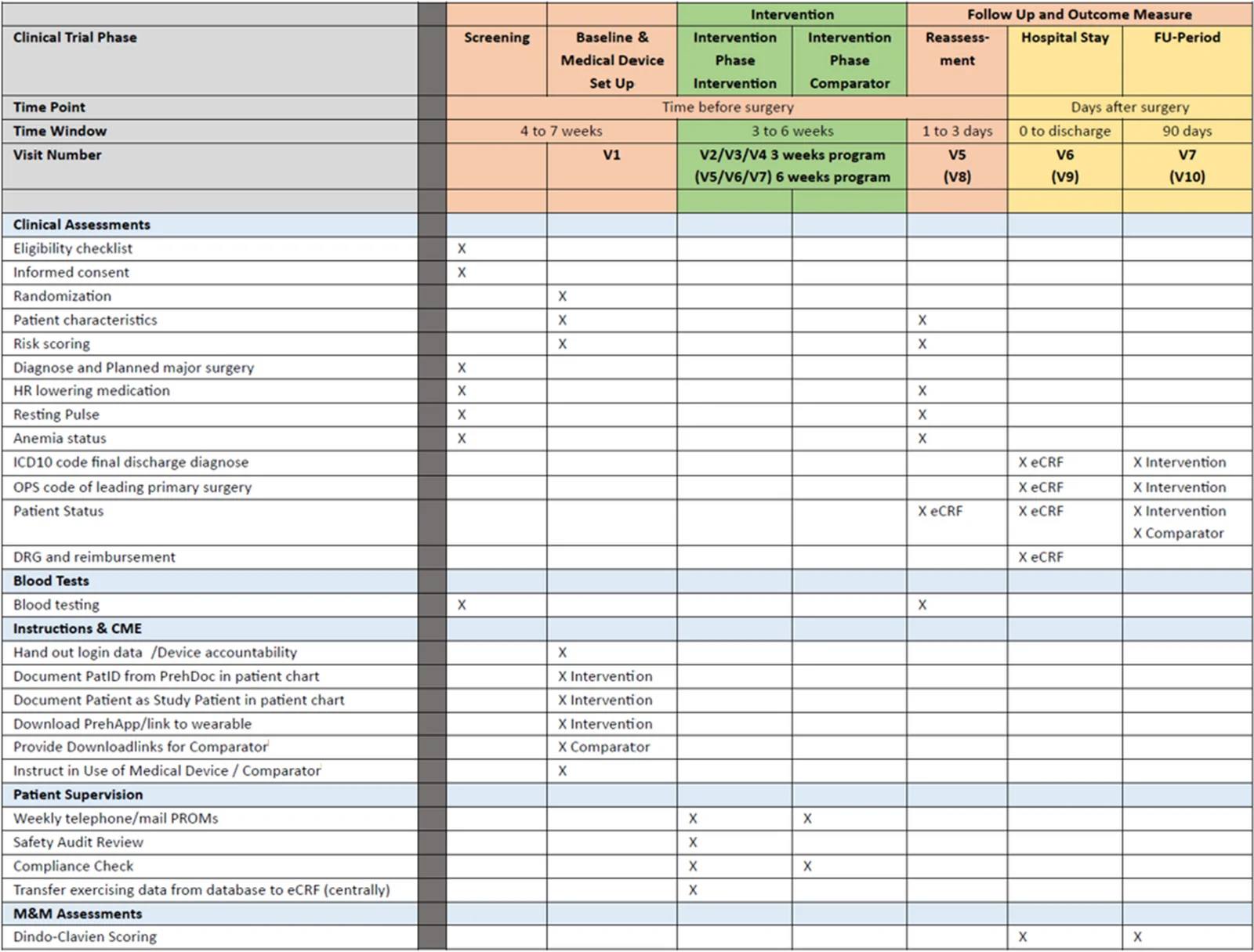
Treatment and visit week scheme. Schedule of enrolment, interventions and assessments of the LUMOS trial

#### Assessments during the follow-up phase

After surgery before discharge, patient status and operative procedure as well as ICD codes and postoperative complications are collected (V6/V9). Ninety days after surgery, patient status as well as Dindo–Clavien scores are evaluated. After surgery, patients will undergo follow-up assessments on days 7 and 90 to evaluate the occurrence of any (serious) adverse events or device failures.

One year after surgery, the patient’s status will be evaluated again. The end of the trial is defined as the last 1-year follow-up of the last patient.

### Criteria for discontinuing or modifying allocated interventions {11b}

Patients can leave the study at any time for any reason if they wish to do so without any consequences.

In case too much data is missing (e.g., missing baseline or all of the follow-up patient-reported outcome measures (PROMs), study visits), the patient will be replaced by a new patient. Early termination or temporary suspension may be recommended exclusively by the DSMB or sponsor following DSMB recommendation in case of unexpected serious safety concerns, unacceptable adverse event patterns, major protocol violations compromising participant safety, or regulatory authority requests such as ethics committee or the BfArM.

Termination criteria: unexpected safety concerns such as a significant increase in frequency or severity of serious adverse events related to the intervention, an unacceptable benefit-risk ratio therefore indication of potential risks of the intervention outweighing its anticipated benefits, a regulatory or ethical requirement due to regulatory, ethical or legal concerns, major protocol violations or GCP violations that compromise participant safety, data integrity or scientific validity, insufficient recruitment or feasibility despite corrective measurements, rendering completion of the study within the planned timeframe scientifically or operationally unfeasible, poor intervention adherence that prevents meaningful evaluation of the primary endpoint despite corrective actions, data integrity concerns, funding or logistical concerns preventing continuation of the study.

#### Device failure

Device failures and risks would be for example lack of safety audit feed although the patient had >2 symptoms during the 6-MWT or during an exercise or although the patient trains outside the defined thresholds (ongoing quality management during the trial by screening the database). Others are lack of exercise transferal into the database, interruption of exercises without a clear reason, or non-storage of data in the database.

#### Reasons to interrupt the trial

An interruption of the trial is used to implement corrective measures and evaluate their effectiveness. If the corrective actions fail, trial termination will be the next step.

All known or foreseeable factors that may impair the clinical trial and its interpretation:

- More than 15% of patients are lost to follow-up
- More than 60% of exercises are not performed
- Generally bad recruitment
- DSMB concerns during the trial
- Lack of financing or budget issues

The first 3 factors are associated with penetration and acceptance of the clinical trial. As it is a new digital therapy, this is a challenge. However, the time for digital therapies is now, the study group has repeatedly proven to be able to perform high-quality clinical trials with and without digital therapies, and we have a special approach to recruitment supported by the study group. There may of course be a situation in which the digital therapy in the interventional groups ends signals that tell us to stop or require an adjustment of the protocol. This is completely unforeseeable currently. However, as we have already performed a sound pilot trial, we do not expect critical deviations from our risk-benefit statement. Budgeting and financing may be critical if we have a major deviation and requirement to adapt the protocol.

Strong reasons to terminate the trial at individual sites are:

- A significant misconduct of the PI at a specific site
- Repeated violations and significant non-compliance with the protocol at specific sites that do not improve even after preventing and correcting actions including team training
- Missing ongoing documentation in the eCRF
- Missing vigilance reporting from the site to the sponsor and authorities
- The fact or suspicion that there is an unacceptable high risk that cannot be mitigated by any measure or intervention of risk by the risk management team into an acceptable risk. This includes an unacceptable health risk or the advisory of the IRB or any regulatory body involved.

In case of an interruption, the sponsor guarantees to provide resources for the follow-up of the patients in the trial as planned. Study participating centers and patients will immediately be informed by the sponsor (main investigator) about the interruption/termination. The sponsor will further inform the IRB and regulatory authorities.

### Strategies to improve adherence to interventions {11c}

We conduct the study in a window of opportunity for life changes in patients before surgery, which should lead to high compliance. Furthermore, the gamification via app use and vibration/colorful display of correct heart frequency ranges should improve adherence. There is a hotline to the treating hospital for every patient.

Measures to enhance adherence:

- Clear planning of the stepwise approach in the Clinical Evaluation Plan
- Study group support from the sponsor to have smooth onboarding and establishment of screening, recruiting, and compliance within the study (reduce barriers approach)
- Multiple telephone/mail contacts with the trial participants
- Safety by design and convincing usability of the devices
- Low documentation workload for the centers considering the items required in the eCRF
- Provision of a dedicated quality management and risk management group

### Relevant concomitant care permitted or prohibited during the trial {11d}

All other necessary therapies for enrolled patients, for example, neoadjuvant therapy, are allowed if the inclusion criteria are met.

### Provisions for post-trial care {30}

After surgery, patients will undergo follow-up assessments on days 7 and 90 to evaluate the occurrence of any (serious) adverse events. One year after surgery, the patient’s status will be evaluated again. The end of the trial is defined as the last 1-year follow-up of the last patient.

Trial-related harm will be covered by a study-specific insurance policy. Safety protocols embedded in The Prehab App will address foreseeable risks, with safety issues monitored, documented, and reported as necessary. The insurance was purchased at HDI Global SE (Nr. 570 10320/03014/03537) with a coverage of 500,000€ per participant and a maximum coverage of 50,000,000€ for all participants in the clinical investigation.

Patients will all be treated in accordance with the local standard after the trial has been terminated. There is no further requirement to specifically adapt after treatment to the trial-specific treatment. All data will be stored in the eCRF as outlined in the Appendix of the documents. The data collected with the app will be stored in an ISO 27001-certified cloud of a server provider located in Germany. The eCRF data will be stored in the Secutrial ® eCRF system which is a regulatory-cleared provider with servers in Germany.

### Outcomes {12}

#### Descriptive measures, survival analyses, and cost analyses

Primary outcome: occurrence of any postoperative complication within 90 days after surgery according to the Clavien–Dindo classification (grades I–V).

Secondary outcomes: 1-year overall survival, 6-min walking distance, QLQ-C30 quality of life score, adherence and compliance, device failures, length of stay, days on ICU/IMC, readmission rates, hospital costs.

There will be an intention to treat analysis of all included patients fulfilling the in- and exclusion criteria. Data will undergo descriptive evaluation and assessment for normal distribution utilizing the Kolmogorov–Smirnov test with Lilliefors correction. Results will be presented as median or mean with standard deviation and interquartile ranges, and/or odds ratios with 95% confidence intervals, where applicable. Survival data will be computed as the time interval from the date of surgery to the date of death, or until censoring if patients are alive at their last visit/contact.

An intention-to-treat analysis will encompass all enrolled patients meeting the predetermined inclusion and exclusion criteria. Additionally, a per-protocol analysis will be conducted.

Compliance analysis in the intervention group includes the analysis of the number of exercises performed and the exercising within the predefined thresholds. We do expect a total of 191 6-MWTs delivering 65,000 pulse points and approximately 53 Mio. pulse points from 191 patients performing 10 exercises of 47 min each during the program that will define compliance as outlined in the following. Compliance of patients in the intervention group will be categorized according to the app algorithm (green: >75% of exercises performed and >75% of all measured values are green or yellow, yellow: 50–75% of exercises performed and 50–75% of all measured values are green or yellow, red: <50% of exercises performed and <50% of all measured values are green or yellow). Comparative compliance in the control group and the intervention group will be based on a descriptive comparison of the weekly safety calls in each group during the interventional phase as PROM analysis.

Surgical procedures and indications will be categorized based on OPS and ICD-10 codes. Subgroup analyses will be conducted using chi-square testing, *T*-testing, Kaplan–Meier survival analysis, and multivariate regression analysis.

Costs will be derived from data recorded in the electronic case report form (eCRF). Costs for a day on ICU are 1500€/day, for a day on the IMC 1000€, and on general ward 500€. The DRG generated by each case will be subtracted from the sum on each treatment unit and the final sums compared between the arms. The cost analysis will be conducted from the hospital/provider perspective based on in-hospital resource utilization including ICU, IMC, and general ward stay.

#### Translational substudies

In the setting of the LUMOS trial, translational substudies are included addressing the value of DNA, RNA, inflammatory markers, and possible other determinants for treatment outcomes. A fresh specimen of (tumor and) fatty (intraabdominal and subcutaneous) tissue should be preserved from every patient for secondary analysis in translational side projects to clarify the mechanisms and effects of exercising before surgery. Therefore, patient blood samples are drawn and analyzed pretherapeutically and during treatment. Formalin-fixed paraffin-embedded (FFPE) tumor specimens are centrally biobanked and analyzed. The biological substudies of the trial are designed for the validation of existing hypotheses and the discovery and generation of new hypotheses addressing important questions in prehabilitation and exercise research.

### Participant timeline {13}

See Table 1 and information on the intervention description.

### Sample size {14}

#### Sample size and power calculation

Based on findings from the literature and our pilot trial “Protego Maxima,” we observed an approximate complication rate or morbidity, as per the Dindo–Clavien classification, of 60% up to 90 days post-surgery [18]. Evidence from randomized-controlled trials suggests a relative reduction in morbidity due to prehabilitation ranging from 30 to 50% [19–21]. Hence, we estimated a clinically meaningful and feasible reduction to a complication rate (Clavien–Dindo I–V) of 45% at day 90 post-surgery in the intervention arm compared to the control arm. Potential center-related heterogeneity may influence the observed effect size. This will be addressed analytically by adjusting for center effects in the statistical models.

Given a study power of 80%, significance set at *p* < 0.05, and an anticipated dropout rate of 10%, we calculated a required sample size of 173 patients per treatment arm. We accounted for a dropout rate of 10% in our calculations. Utilizing the chi-square test and probability calculations in two groups led to a sample size of 191 patients per treatment arm (382 patients in total, including dropouts), in accordance with methods described by Rosner [22].

### Recruitment {15}

Patients will be recruited at all outpatient clinics of the enrolled centers. They are all university hospitals or maximum care centers, all undertaking several hundred major surgeries each year.

## Assignment of interventions: allocation

### Sequence generation {16a}

The patients will be randomized after eligibility confirmation, which will in most cases also be the baseline assessment day. The randomization sequence is computer-generated by an independent statistician using randomly varying block sizes and stratified by study center and diagnostic/surgical category. The allocation list is generated before study initiation and securely stored. For equal distribution of indications, sex, age, and center effects, we will create specific randomization blocks for the trial. The centers will receive envelopes with randomization information. The patients will be randomized during baseline assessment using block randomization stratified by study center and diagnostic/surgical category. This approach ensures balanced allocation of patients between treatment arms within each participating center and accounts for potential center-specific differences in surgical practice and perioperative care.

### Concealment mechanism {16b}

Allocation concealment is ensured using sequentially numbered, sealed envelopes prepared according to the computer-generated randomization list. Envelopes are opened only after written informed consent and completion of eligibility criteria and baseline assessments. Due to the nature of the intervention, participants and investigators are not blinded after allocation.

### Implementation {16c}

After signing the informed consent form, the randomization of the patient will be revealed by opening the randomization envelope and the group will be revealed to the patient and the researcher.

## Assignment of interventions: blinding

### Who will be blinded {17a}

Patients, researchers, and surgeons will not be blinded.

### Procedure for unblinding if needed {17b}

The trial design is open label; therefore, there is no unblinding procedure.

## Data collection and management

### Plans for assessment and collection of outcomes {18a}

#### Data management and data safety

All clinical data will be securely stored in the electronic case report form (eCRF) utilizing the SecuTrial® System, a web-based data management application. SecuTrial® includes automated range checks, completeness checks, consistency rules, audit trails, user-right management, electronic query handling, and validation procedures to ensure data quality before database lock. Data will be retrieved from the electronic health records of the patients and from the information entered into the application. Data entered will be stored on server systems compliant with DIN ISO 27001, located in Germany. The cloud computing regulation of the Bundesinstitut für Sicherheit in der Informationstechnologie (BSI, National Institute for Cybersecurity) C5 will be complied with. Only authorized and trained members of the study team will have access to enter data, systematically ensuring accuracy and completeness. Investigators responsible for postoperative complication assessment will receive centralized training in the application of the Clavien–Dindo classification to ensure consistent complication grading across centers. Clinical data will be pseudonymously documented using specific identification numbers. Participants will remain anonymous in all study reports, with no identifiable personal information disclosed. Study data will be integrated into statistical software and analyzed by the Institute of Biostatistics and Mathematical Modeling at Frankfurt.

### Plans to promote participant retention and complete follow-up {18b}

The patients will receive extensive information about the study setup and requirements during the recruitment. The importance of completion of the follow-up will be stressed. Patients are allowed to stop at any time during the study and are not obliged to give a reason to discontinue. Usage of the application is done remotely so at the convenience of every patient. Throughout the follow-up period, the researchers will check responses and if necessary, contact patients for completion of their follow-up.

### Data management {19}

All clinical data will be securely stored in the electronic case report form (eCRF) utilizing the SecuTrial® System. (S)AEs will be recorded in the eCRF. Backups on the protected research server will be made regularly (once per 3 months). Informed consent and end-of-trial dates will be recorded in the electronic patient dossier and signed paper forms will be stored within our hospital in a locked room.

#### Data monitoring

Regular monitoring will be conducted by the data monitoring committee following Good Clinical Practice guidelines and DIN ISO 14155 standards. Monitors will review data for protocol compliance and accuracy, ensuring alignment with source data documents such as electronic health records and The Prehab App. Monitoring activities will guarantee completeness of data entry, accuracy of study management, and documentation of safety measures.

Original data will be printed, transferred into the eCRF, and kept in the ISF as original at each site for 15 years.

### Confidentiality {27}

The study will adhere to the Declaration of Helsinki and Good Clinical Practice standards outlined in DIN ISO 14155, Medical Device Regulation, and the Medical Devices Implementation Act. All documents will be stored safely in confidential conditions following the ISO 27001 and ISO14155 requirements for safe data storage. On all study-specific documents, other than the signed consent, the participant will be referred to by the study participant number/code, not by name. The trial staff will ensure that the participants’ anonymity is maintained. The participants will be identified only by initials and a participant’s ID number on the CRF and any electronic database. All documents will be stored securely and will be only accessible by trial staff and authorized personnel. The study will comply with the Data Protection Act 1998 which requires data to be anonymized as soon as it is practical to do so; the key to the identification code list will only be available to the research team during the study and will be documented and safeguarded by the principal investigator according to research guidelines after completion of the study. No patient identification details will be reported in publications.

### Plans for collection, laboratory evaluation, and storage of biological specimens for genetic or molecular analysis in this trial/future use {33}

Blood samples will be collected upon trial inclusion and preoperatively. Laboratory evaluations will be conducted according to general evaluations in the participating hospitals after SOP. Biological sampling is done according to the standards of the UCT in the general bio database. Formalin-fixed paraffin-embedded (FFPE) tumor specimens are centrally biobanked and analyzed. They will be used in future studies.

## Statistical methods

### Statistical methods for primary and secondary outcomes {20a}

Data will be analyzed using IBM SPSS (version 29 or more current version, if applicable). Surgical procedures and indications will be categorized based on OPS and ICD-10 codes. Subgroup analyses will be conducted using chi-square testing, *T*-testing, Kaplan–Meier survival analysis, and multivariate regression analysis. The center will be included as a stratification variable. Data will be descriptively evaluated and tested for normal distribution with the Kolmogorov–Smirnov test with Lilliefors correction. Data will be expressed as median or means with standard deviation and interquartile and/or odds ratios with 95% confidence intervals where appropriate. Survival data will be calculated as time intervals from the date of their death to the date of operation, respectively be censored, if they are alive at their last visit/contact. Subgroup analyses will be conducted for predefined categories including cancer versus non-cancer surgery, abdominal versus non-abdominal procedures, and higher-risk versus lower-risk patients. Surgical category will additionally be included as a covariate in multivariable analyses to account for potential heterogeneity in baseline risk. The primary analysis will follow the intention-to-treat principle. Multivariable regression models will be used to assess treatment effects while adjusting for study center and relevant baseline characteristics. Where appropriate, mixed-effects regression models including study center as a random effect will be applied to account for the multicenter study design.

Costs will be derived from data recorded in the electronic case report form (eCRF). Costs for a day on ICU are 1500€/day, for a day on the IMC 1000€, and on general ward 500€. The DRG generated by each case will be subtracted from the sum on each treatment unit and the final sums compared between the arms. This is a two-sided approach using *T*-testing of two independent groups and a *p *value < 0.05 is regarded clinically significant.

The 95% confidence interval of the fixed effect size will be used to assess whether the treatment difference reaches the minimally clinically important difference.

All (S)AE(I)s will be summarized and recorded including the nature, date and time of onset, date of resolution, determination of seriousness, severity, action taken, outcome, and possible causality to study treatment. SAE data will be presented in a descriptive manner.

### Interim analyses {21b}

The sponsor will set up a trial DSMB that will receive the quarterly safety update reports and analyze them accordingly for risks and benefits throughout the trial. In accordance with the statistical analysis plan, we will provide interim analysis to detect early signals of success or failure in the clinical trial. The study composes of a low-risk digital interventional trial; therefore, no formal interim efficacy analysis with predefined statistical stopping boundaries is planned. However, safety and trial conduct will be reviewed regularly. According to the DSMB’s safety reports, internal and external monitoring, and audits, there might be protocol modifications, temporary suspension, or early termination in the event of relevant safety concerns, unacceptable adverse event patterns, or major concerns regarding trial feasibility or integrity. Stopping rules were predefined within the ethics application.

### Methods for additional analyses (e.g., subgroup analyses) {20b}

Subgroups will be analyzed according to risk grouping in both groups, and compliance levels comparing the intervention and the control group based on the PROM analysis. Multivariable analysis with baseline risk factors (modifiable in both groups for later application), adjusting for levels of health literacy, risk grouping, and operations, will be performed. Significance levels of 0.05 will be assumed as clinically meaningful. A two-sided test will be applied. Center and subgroup effects will be interpreted with respect to multiplicity.

### Methods in analysis to handle protocol non-adherence and any statistical methods to handle missing data {20c}

The primary outcome will be assessed using an intention-to-treat analysis. Missing data will be reduced to a minimum by using the appropriate measures described above. Patients whose data are missing will be treated in accordance with the intention to treat principle. As the app does not allow to proceed when critical data is missing, the probability of this event is very low.

In general, it is strictly forbidden to not adhere to the clinical trial protocol outlined in the protocol. Deviations from the original statistical analysis plan can be recommendations from the DSMB or the regulatory authorities. Potential reasons can be justifiable in case patients need to be protected from potential harm in emergency situations. These situations will be documented and immediately reported to the independent review board and to the competent authority in analogy with the SAE reporting procedures. In addition, a risk-benefit evaluation will be made for those events and for the whole study cohort based on DSMB recommendations. Based on the conclusion, an amendment will be prepared, the study will be carried on, or the study will be stopped. Preventing actions include a dedicated and structured onboarding for all investigators involved. This will be documented on trial logs and stored in the ISF and TMF. Study procedures will be supported by clear checklists and supporting material for the investigators.

### Plans to give access to the full protocol, participant-level data, and statistical code {31c}

The results of the study are going to be published in a peer-reviewed journal; the data will be anonymized. The datasets used and/or analyzed during the current study can be made available by the corresponding author upon reasonable request.

## Oversight and monitoring

### Composition of the coordinating center and trial steering committee {5d}

Direct access will be granted to authorized representatives from the sponsor, the funding source, the monitors, Capreolos GmbH Knowledge and Quality, Safety and Risk Management Department, the host institution, and the regulatory authorities to permit trial-related monitoring, audits, and inspections.

Principal investigator: takes supervision of the trial and medical responsibility of the patients. The PI oversees day-to-day safety of the trial. Study coordinator: trial registration, coordinates study, visits, and annual safety reports. Data manager: organizes data capture, safeguards, quality, and data. Study physician: identifies potential recruits, takes informed consent, and ensures follow-up according to protocol. The study team meets online weekly. All parties at a center enter data into the eCRF. The coordinating center will ensure safety and data quality as well as the monitor at each site. The coordinating center is responsible for the overall conduct of the trial, including study coordination, data management, and safety reporting. The principal investigator provides overall scientific leadership and clinical oversight. The Project Management Group manages the day-to-day operational aspects of the study, including recruitment, monitoring of protocol adherence, and coordination between participating centers. The group meets weekly to review study progress.

Safety oversight is provided by the Data Safety Monitoring Board (DSMB), which reviews quarterly safety reports and provides recommendations regarding trial continuation and safety monitoring.

### Composition of the data monitoring committee, its role and reporting structure {21a}

The sponsor will set up a trial DSMB that will receive the quarterly safety update reports and analyze them accordingly for risks and benefits throughout the trial. In accordance with the statistical analysis plan, we will provide interim analysis to detect early signals of success or failure in the clinical trial.

### Adverse event reporting and harms {22}

#### Adverse events

All adverse events (AEs), adverse device effects (ADEs), serious adverse events (SAEs), and serious adverse device effects (SADEs) occurring during the prehabilitation or subsequent follow-ups will be documented in the electronic case report form (eCRF). An AE is characterized as any undesirable sign, symptom, or disease temporally associated with the application of the device, without necessitating a causal relationship. If a potential causal relationship with the device is conceivable, the AE is classified as an ADE. For all AEs and ADEs, the duration, consequences, and assessment of their relationship to the device will be recorded. AEs or ADEs resulting in life-threatening illness, injury, extended hospitalization, permanent disability, necessitating medical or surgical intervention, or death will be designated as SAEs or SADEs. They will be promptly reported to the sponsor, legal representative, and manufacturer in accordance with the regulatory obligations of the Medical Device Regulation (MDR) and the Medical Device Law Implementation Act (MPDG). Appropriate follow-up actions will be undertaken as required.

The reporting of SAEs and device deficiencies will be carried out in accordance with the Regulation (EU) 2017/745 (MDR) from May 26, 2021, in conjunction with the Medical Device Law Implementing Act (“Medizinprodukterecht-Durchführungsgesetz,” MPDG) for all clinical investigations that must be applied for or notified to the federal higher authority (“Bundesoberbehörde”). For all reportable events according to Article 80 MDR or § 64 paragraph 1 MPDG which indicate an imminent risk of death, serious injury, or serious illness and that requires prompt remedial action for other patients/subjects, users, or other persons or a new finding to it: immediately, but not later than 2 calendar days after awareness by the sponsor of a new reportable event or of new information in relation with an already reported event. This includes events that are of a significant and unexpected nature such that they become alarming as a potential public health hazard. It also includes the possibility of multiple deaths occurring at short intervals.

For SAEs or device deficiencies (DD) from German trial sites, the SAE/DD individual notification form of the BfArM will be used and sent to mpsae@bfarm. The sponsor will use the individual SAE report form of the BfArM. In addition, a quarterly summary of the SAE assessment will be performed using the Evaluation form and Annex 3.1 complication rates provided by the BfArM. This includes all reportable SAEs/DDs and in addition, SAEs and DDs that are included in endpoints according to the CIP (Clinical Investigation Plan). The data will be presented in comparison to scientific data from literature or other sources such as the risk analysis for all serious complications that may be related to the investigational medical device, including adverse events as a complication rate analysis. All reports will be sent to MPSAE@bfarm.de. All required procedures can be assessed in German and English language under https://www.bfarm.de/EN/Medical-devices/Applications-and-reports/SAE-report/Clinical-investigation/_node.html and will be implemented for the LUMOS trial in the SOPs for vigilance reporting by the sponsor. Reporting will be performed once every 3 months starting from the first patient being included in the clinical investigation. The following data will be provided to the competent authorities and the IRB will be informed about the regular safety reporting.

### Frequency and plans for auditing trial conduct {23}

Details and amount of monitoring are in general defined by the risk category according to the Human Research Act and confirmed by the SCTO-“Monitoring Risk Analysis.” Oversight of the trial conduct is provided through regular monitoring according to the Good Clinical Practice and ISO 14155 standards. Further criteria (such as the experience on-site, the use of a validated database, and function as the coordinating site) could increase or decrease the extent of monitoring for a clinical investigation. For the LUMOS trial, a risk category of low to medium risk was defined. Every site needs a site initiation visit that includes structured training in documents. The training can be done remotely. One hundred percent of informed consent needs to be checked. The first visit should take place after 1–2 patients at sites that plan to include less than 20 patients and after 5 patients at larger sites. A random list of approximately 80 patients (1 for each 5 randomized at each center) will define the patients that require monitoring of key data. This accounts for >20% of all patients that were included. In case there are repeated problematic findings at a site, the sponsor can increase the number of monitoring of key data of the clinical investigation to 50%. There shall be 1 visit per year at unproblematic sites or sites with less than 20 patients and 2 visits per year at problematic sites or with more than 20 patients. Queries by the monitor will be done via eCRF structure. Each monitoring visit will include a monitoring report with a response by the project management group addressing each finding and query by the monitor. Monitoring is done approximately every month, depending on recruitment numbers. A close-out visit will be performed at each site. This can be done remotely. For more information, the monitoring plan can be consulted. The Project Management Group is responsible for daily trial conduct and meets weekly. Internal auditing according to the standard operating procedure of the central study coordination will be conducted twice a year. All CAPAs will be written down. Vendor audits and external audits will take place at least once a year. Auditing can also take place by national or international health authorities.

### Plans for communicating important protocol amendments to relevant parties (e.g., trial participants, ethical committees) {25}

In case of the necessity of an amendment, we will adhere to the CAPA SOP in the DIN ISO14155 compliant clinical trials management. All changes will be documented in the document history. We use versions. For a significant amendment, we will go from, e.g., version 1 to 2 or 3, etc. For a minor amendment, we will go from, e.g., version 1 to version 1.1 or 1.2, etc. Reasons for an amendment can be recommendations by the DSMB, requests by authorities, or changes can be necessary because of vigilance signals or internal/external audits that may occur during the trial. The CAPA process will define if it is a significant major or minor amendment. The clinical trial will be continued with the old protocol in case of a minor protocol amendment until the new protocol version has been approved or the clinical trial will be set on hold in case there is a major safety-relevant amendment to the CIP. All substantial amendments will be notified to the competent authority. If amendments affect participants in any way, they are informed about the changes. If needed, additional consent will be requested and registered. Also, online trial registries will be updated accordingly. All substantial protocol amendments will first be reviewed and approved by the sponsor and relevant authorities/ethics committees, after which participating centers will be informed by the principal investigator. Updated protocol versions will be filed in the Investigator Site File. Protocol deviations will be documented in a breach form, and trial registry entries will be updated where applicable.

### Patient and public involvement

Formal patient and public involvement in the protocol design was limited, but patient-centered aspects of the intervention were informed by prior usability and feasibility work with the app and by the inclusion of patient-reported outcomes. Furthermore, there has been a dedicated peer analysis of all stakeholders that were published elsewhere as well as a usability trial in potential patients and patients as well as other stakeholders that showed excellent usability of our solution that is now tested in its final intended use [23].

### Dissemination plans {31a}

The study protocol and the results of the study will be reported in scientific journals. The results of this research will be disclosed completely in international peer-reviewed journals. Both positive and negative results will be reported. The publication policy should cover authorship, acknowledgments, and review procedures for scientific publications. Participants will be informed about study results upon request.

## Discussion

Despite advancements in surgical care, mortality rates among surgical patients remain substantial, accounting for 8% of all hospital deaths [2]. While improvements in patient selection, perioperative protocols such as ERAS, surgical techniques, and multimodal treatment strategies have been made, there is still a pressing need for further enhancement [24]. The perioperative phase, including the period before elective surgery or during neoadjuvant therapy, presents an opportunity to optimize patient outcomes [25]. Identifying and addressing preoperative modifiable risk factors is crucial in this regard, with robust risk scoring systems being integral tools in risk assessment. The risk calculator integrated into The Prehab App utilizes scientifically validated risk scoring systems to tailor prehabilitation strategies for individual patients [26–29].

While some studies have demonstrated improvements in postoperative outcomes with prehabilitation, there remains considerable heterogeneity in study designs, limiting comparability and complicating the implementation of prehabilitation programs in clinical practice [12, 30]. Additionally, the impact of prehabilitation on mortality outcomes remains debated, with conflicting findings across studies [31, 32].

Prior studies as the “Protego Maxima” trial have underscored the potential of digital tools, such as “The Prehab App,” in risk screening and preoperative optimization. Evidence-based tools embedded within the app have shown promising correlations with short-term surgical outcomes, enhancing risk prediction and informing prehabilitation strategies [29].

The present study seeks to address these challenges by evaluating the effectiveness of a digital prehabilitation program and by developing a safe and valid medical device for reducing perioperative morbidity and mortality. In an era marked by pandemics, fast lifestyles, and strained healthcare systems, the need for remote, patient-centered interventions is paramount. The digital nature of the program allows for flexibility in usage, empowering patients to engage in prehabilitation from the comfort of their homes. The incorporation of objective assessments alongside patient-reported outcomes reflects the growing emphasis on holistic patient care in research.

Despite the promise of prehabilitation, divergent study outcomes underscore the need for further investigation and validation. The current trial aims to fill this gap by rigorously evaluating a digital individual prehabilitation program’s efficacy and by offering high-quality data by considering multiple surgical departments and multicentric high-volume centers. In the context of major surgical procedures, high-quality and meaningful data equate to mitigated harm resulting in enhanced quality of care. A key indicator of this is a reduction in 90-day postoperative morbidity among surgical patients. Analysis of postoperative days spent in normal wards, Intermediate Care Units (IMC), and Intensive Care Units (ICU) may provide insights into the severity of postoperative illness, with potential implications for long-term outcomes.

Digital healthcare applications offer a promising avenue for home-based interventions, enhancing patient engagement and accessibility. However, the availability of validated prehabilitation apps remains limited, highlighting the need for continued development and validation in this field.

### Limitations

There are some limitations to be considered. First, prehabilitation as understood in this study with the focus on bodily training excludes patients before cardiac surgery as well as trauma surgery patients. We do include all other surgical patients in this study, though. Furthermore, complication rates may vary across centers and surgical categories due to differences in surgical case mix and perioperative care pathways. Although the sample size calculation was based on previously published complication rates, potential center-related heterogeneity may influence the observed effect size. This will be addressed analytically by adjusting for center effects in the statistical models. Second, the follow-up period after surgery is relatively short (1 year) and long-term efficacy remains to be investigated. There might occur performance bias through different perioperative care and detection bias for subjective outcomes, but this should be negligible via training of study members and standard operating procedures. Lastly, only patients in possession of a smartphone can participate in this trial. These days, non-possession should be a rare occurrence.

## Trial status

Recruitment for participants has started in December 2025. The trial is expected to conclude by December 31, 2026. The current protocol is Lumos Clinical Trial Protocol V1.3; June 7, 2024.

## Supplementary Information

Supplementary Material 1.

Supplementary Material 2.

## Data Availability

The datasets used and/or analyzed during the current study will be made available from the corresponding author upon reasonable request and anonymizing the dataset accordingly.

## Abbreviations

6-MWT: 6-Min walking test
(S)AE: (Serious) adverse event
AEs: Adverse events
ADEs: Adverse device effects
API: Application Programming Interface
BfArM: Federal Institute for Drugs and Medical Devices (Bundesinstitut für Arzneimittel und Medizinprodukte)
BMI: Body mass index
BSI: Bundesinstitut für Sicherheit in der Informationstechnik (Federal Office for Information Security)
C5: Cloud Computing Compliance Criteria Catalog
CAPA: Corrective and Preventive Action
CIP: Clinical Investigation Plan
DD: Device deficiency
DIN: Deutsches Institut für Normung (German Institute for Standardization)
DNA: Desoxyribonucleic acid
DRG: Diagnosis-Related Group
DSMB: Data Safety Monitoring Board
eCRF: Electronic case report form
ERAS: Enhanced Recovery After Surgery
FFPE: Formalin-fixed paraffin-embedded
HR: Heart rate
IBM: International Business Machines
ICD: International Classification of Diseases
ICD-10: International Classification of Diseases, 10th Revision
ICU: Intensive Care Unit
IMC: Intermediate Care Unit
IRB: Institutional Review Board
ISO: International Organization for Standardization
ISF: Investigator Site File
MDR: Medical Device Regulation
MPDG: Medical Device Law Implementation Act (Medizinprodukte-Durchführungsgesetz)
OPS: Operation and Procedure Classification System
PROM: Patient-reported outcome measures
QLQC-30: Quality of Life Questionnaire Core 30
REC: Research Ethics Committee
RNA: Ribonucleic acid
SAE: Serious adverse event
SADEs: Serious adverse device effects
SAEs: Serious adverse events
SOP: Standard operating procedure
SPSS: Statistical Package for the Social Sciences
UCT: University Cancer Center
V2-V7: Visit 2 to visit 7
V5/V8: Visit 5/visit 8
V6/V9: Visit 6/visit 9
