# Supplementary material for: Study protocol for a multicenter prospective randomized-controlled trial to test a systematic remote and app-based prehabilitation program in comparison to education-only before major surgery: the LUMOS Study
Source: Trials. 2026 Sep 10;27:567. doi: 10.1186/s13063-026-09977-w (PMC13560359; doi:10.1186/s13063-026-09977-w)
Supplement: Supplementary file 1 [file 13063_2026_9977_MOESM1_ESM.docx]

**Supplemental materials**

| 1. | 5-070 to 5-073, 5-077 (endocrine glands) |
| --- | --- |
| 2. | 5-314, 5-316, 5-32 to 5.334, 5-339 to 5-349 (trachea, lung and bronchus) |
| 3. | 5-38 to 5-39 (vascular surgeries without cardiovascular surgery) |
| 4. | 5-404.1 to 5-405, 5-406.2 to 5-406.9+a+b, 5-407 (lymphadenectomies) |
| 5. | 5-413 and 5-419 (splenectomy) |
| 6. | 5-423 to 5-427 (esophagectomy) |
| 7. | 5-434 to 5-438 (gastrectomy) |
| 8. | 5-445 and 5-446 (non-resecting gastric bypass operations) |
| 9. | 5-454 (small bowel resections) |
| 10. | 5-455 and 4-466 (large bowel resections including Hartmann’s revision) |
| 11. | 5-456 (proctocolectomy) |
| 12. | 5-484 and 5-485 (rectum resections) |
| 13. | 5-502 and 5-503 (anatomical liver resection) |
| 14. | 5-512, 5-515, 5-518.4 and .5, 5-519 (biliary tract resections) |
| 15. | 5-523, 5-524, 5-525, 5-527 and 5-529 (pancreas resections and draining procedures) |
| 16. | 5-536, 5-538, 5-539 (abdominal wall hernias) |
| 17. | 5-553, 5-554, 5-575 to 5-579, 5-604 (urologic resections: kidney, bladder, prostate) |
| 18. | 5-652, 5-653, 5-661, 5-682 to 5-687 (gynecologic resections of uterus, ovary, cervix) |
| 19. | 5-87 and 5-88 (breast surgery) |

Table S1 OPS codes in accordance with the DIMDI OPS version 2023: (<https://www.dimdi.de/static/de/klassifikationen/ops/kode-suche/opshtml2023/#code5>)
